# Genome-Wide Analysis of *Corynespora cassiicola* Leaf Fall Disease Putative Effectors

**DOI:** 10.3389/fmicb.2018.00276

**Published:** 2018-03-02

**Authors:** David Lopez, Sébastien Ribeiro, Philippe Label, Boris Fumanal, Jean-Stéphane Venisse, Annegret Kohler, Ricardo R. de Oliveira, Kurt Labutti, Anna Lipzen, Kathleen Lail, Diane Bauer, Robin A. Ohm, Kerrie W. Barry, Joseph Spatafora, Igor V. Grigoriev, Francis M. Martin, Valérie Pujade-Renaud

**Affiliations:** ^1^Université Clermont Auvergne, Institut National de la Recherche Agronomique, UMR PIAF, Clermont-Ferrand, France; ^2^CIRAD, UMR AGAP, Clermont-Ferrand, France; ^3^AGAP, Université Montpellier, CIRAD, Institut National de la Recherche Agronomique, Montpellier SupAgro, Montpellier, France; ^4^Institut National de la Recherche Agronomique, UMR INRA-Université de Lorraine “Interaction Arbres/Microorganismes”, Champenoux, France; ^5^Departemento de Agronomia, Universidade Estadual de Maringá, Maringá, Brazil; ^6^United States Department of Energy Joint Genome Institute, Walnut Creek, CA, United States; ^7^Department of Microbiology, Utrecht University, Utrecht, Netherlands; ^8^Department of Botany and Plant Pathology, Oregon State University, Corvallis, OR, United States; ^9^Department of Plant and Microbial Biology, University of California, Berkeley, Berkeley, CA, United States

**Keywords:** *Corynespora cassiicola*, *Hevea brasiliensis*, plant-pathogens interaction, effectors, cassiicolin, genomics, gene expression

## Abstract

*Corynespora cassiicola* is an Ascomycetes fungus with a broad host range and diverse life styles. Mostly known as a necrotrophic plant pathogen, it has also been associated with rare cases of human infection. In the rubber tree, this fungus causes the *Corynespora* leaf fall (CLF) disease, which increasingly affects natural rubber production in Asia and Africa. It has also been found as an endophyte in South American rubber plantations where no CLF outbreak has yet occurred. The *C. cassiicola* species is genetically highly diverse, but no clear relationship has been evidenced between phylogenetic lineage and pathogenicity. Cassiicolin, a small glycosylated secreted protein effector, is thought to be involved in the necrotrophic interaction with the rubber tree but some virulent *C. cassiicola* isolates do not have a cassiicolin gene. This study set out to identify other putative effectors involved in CLF. The genome of a highly virulent *C. cassiicola* isolate from the rubber tree (CCP) was sequenced and assembled. *In silico* prediction revealed 2870 putative effectors, comprising CAZymes, lipases, peptidases, secreted proteins and enzymes associated with secondary metabolism. Comparison with the genomes of 44 other fungal species, focusing on effector content, revealed a striking proximity with phylogenetically unrelated species (*Colletotrichum acutatum, Colletotrichum gloesporioides, Fusarium oxysporum, nectria hematococca*, and *Botrosphaeria dothidea*) sharing life style plasticity and broad host range. Candidate effectors involved in the compatible interaction with the rubber tree were identified by transcriptomic analysis. Differentially expressed genes included 92 putative effectors, among which cassiicolin and two other secreted singleton proteins. Finally, the genomes of 35 *C. cassiicola* isolates representing the genetic diversity of the species were sequenced and assembled, and putative effectors identified. At the intraspecific level, effector-based classification was found to be highly consistent with the phylogenomic trees. Identification of lineage-specific effectors is a key step toward understanding *C. cassiicola* virulence and host specialization mechanisms.

## Introduction

*Corynespora cassiicola* (Berk. & M. A. Curtis) is an Ascomycetes fungus responsible for diseases in a wide range of plants (Farr and Rossman, 2016), mainly in tropical and subtropical areas or greenhouses. It has also been found in nematodes (Carris et al., 1986), sponges (Zhao et al., 2015) and in rare cases of human infections (Mahgoub, 1969; Huang et al., 2010; Yamada et al., 2013; Yan et al., 2016). In the rubber tree, *C. cassiicola* causes the *Corynespora* leaf fall (CLF) disease, characterized by necrotic lesions on the leaves and massive defoliation in susceptible cultivars. CLF was first observed in Sierra Leone (Deighton, 1936). Initially limited to nurseries, CLF was recognized as a threat to rubber farming after a severe outbreak in Sri Lanka in the late 1980's, which led to the uprooting of more than 4,000 ha of the highly susceptible rubber cultivar RRIC103 (Liyanage et al., 1986). Since then, CLF has gradually spread to most rubber producing areas in Asia and Africa, impairing natural rubber production yields (Kuruvilla Jacob, 2006).

*Corynespora* is a polyphyletic genus in the large Pleosporales order, mostly composed of plant pathogens. *C. cassiicola* forms with *Corynespora smithi* a specific family within Pleosporales (Schoch et al., 2009). Like most species of the *Corynespora* genus, *C. cassiicola* is anamorphic.

*Corynespora cassiicola* isolates display various life styles, from endophyte (Collado et al., 1999; Gond et al., 2007; Promputtha et al., 2007; Suryanarayanan et al., 2011; Déon et al., 2012b) to saprophyte (Kingsland, 1985; Lee et al., 2004; Cai et al., 2006), but are mostly reported as necrotrophic pathogens.

The high genetic diversity of the species is largely documented (Silva et al., 1998, 2003; Atan and Hamid, 2003; Nghia et al., 2008; Dixon et al., 2009; Qi et al., 2011; Déon et al., 2014; Hieu et al., 2014; Shuib et al., 2015). We have previously analyzed the diversity of 129 *C. cassiicola* isolates from various hosts (including 71 from the rubber tree) and of various geographical origins, based on the polymorphism of four combined *loci*, as proposed by Dixon et al. (Dixon et al., 2009). We identified eight major clades (A–H), with no clear geographic or host speciation (Déon et al., 2014).

*Corynespora cassiicola* diversity is not structured by strict host specificities, but rather host specialization (Dixon et al., 2009; Hieu et al., 2014), which suggests the involvement of specialized effectors recognized by a limited range of compatible hosts. In addition, in the rubber tree, *C. cassiicola* virulence profiles vary significantly depending on the cultivars (Breton et al., 2000; Atan and Hamid, 2003; Nghia et al., 2008; Qi et al., 2009; Déon et al., 2014).

The only *C. cassiicola* effector characterized so far is cassiicolin. This necrotrophic toxin was purified from the filtrate of the highly virulent isolate CCP, collected from diseased rubber trees in the Philippines (Breton et al., 2000; de Lamotte et al., 2007). Cassiicolin is a 27 amino acid glycoprotein with six cysteines engaged in three disulphide bonds (Barthe et al., 2007). It is matured from a pre-protein encoded by the three-exon *Cas* gene (GenBank EF667973) (Déon et al., 2012a). *Cas* is transiently upregulated 24–48 h after inoculation of rubber tree leaves with isolate CCP, just before the first symptoms occur in a susceptible cultivar, suggesting a role during the first phase of infection. Various cassiicolin isoforms (Cas1 to Cas6) were later identified by PCR screening of 70 isolates from various hosts and geographical origins. *Cas1* refers to the initially described *Cas* gene, from CCP. We thus adopted a typology based on (i) the genetic group (A-H) and (ii) the toxin class determined according to the *Cas* gene polymorphism (Déon et al., 2014). Isolates of type C/Cas1 were found to be generally more aggressive than those of other types, but further study will be necessary to describe the possible associations between genetic group, toxin class and virulence profiles. Notably, some *C. cassiicola* isolates without *Cas* gene (isolates of toxin class Cas0) were nevertheless found to be moderately virulent in the rubber tree, evidencing effectors other than cassiicolin. Phenotypic diversity among *C. cassiicola* isolates was also evidenced by measuring the leaf damage (electrolyte leakages) caused by the application of fungal culture filtrates (Tran et al., 2016). Both qualitative and quantitative variations among the secreted effectors, together with multiple plant targets or sensitivity factors, may explain the observed phenotypic diversity.

This study set out to identify the putative effectors involved in CLF in the rubber tree, from the genomes of *C. corynespora* isolates representative of the intraspecific diversity. The genome of the virulent reference isolate CCP was first sequenced, assembled, and annotated by the US Department of Energy Joint Genome Institute (JGI) as part of the 1,000 Fungal Genomes Project (Grigoriev et al., 2014). Putative effectors were predicted and compared with the effector repertoires of 44 other fungal species. Transcript profiling was conducted to identify the functional effectors differentially expressed during a compatible interaction with the rubber tree. Lastly, we sequenced and assembled 35 genomes of new *C. cassiicola* isolates to compare their respective effector repertoires. Our findings are discussed in relation to the phylogeny and known physiological specificities of the selected isolates, with a special focus on the cassiicolin effector.

## Materials and methods

### Biological material

*Corynespora cassiicola* (Berk. & M.A. Curtis) isolate CCP, highly virulent in the rubber tree (Breton et al., 2000), was chosen as phytopathogenic reference for the species. Genomic DNA and RNA samples were prepared from CCP mycelium grown for 3 weeks in liquid Czapeck medium, at 26/25°C (day/night), 12/12h light cycle. This culture was set up from 7-day-old cultures grown on PDA medium (potato dextrose agar, DIFCO), at 26/25°C (day/night), in the dark. For long-term conservation, mycelium plugs were kept at −80°C in 20% glycerol.

Interspecies comparative genomic analysis involved the CCP genome (http://genome.jgi.doe.gov/Corca1/Corca1.home.html) along with 44 other fungal genomes available from the JGI MycoCosm Portal (Table S1). Genome assembly from UM591, *C. cassiicola* isolate of human origin, was obtained from the NCBI Whole Genome Shotgun project under the accession http://www.ncbi.nlm.nih.gov/nuccore/JAQF00000000.1.

Intraspecies comparative genomic analysis was carried out on CCP along with 35 other *C. cassiicola* strains previously isolated from various host plants (Déon et al., 2014; Table 1) with CCP as reference isolate.

**Table 1:** **(A)** Metrics of the CCP genome assembly **(B)** Repetitive DNA content and transposable elements.

**A**		
**Genome assembly**		
Genome Assembly size (Mbp)	44.85	
Sequencing read coverage depth	58.8x	
No. of contigs	500	
No. of scaffolds	244	
No. of scaffolds > = 2Kbp	173	
Scaffold N50	8	
Scaffold L50 (Mbp)	2.51	
No. of gaps	256	
% of scaffold length in gaps	0.3%	
Three largest Scaffolds (Mbp)	4.76, 2.92, 2.67	
Assembled_RNAseq_reads	98.5%	
No. of gene models	17,167	
**Gene models**	**Average**	**Median**
Gene length (bp)	1,610	1,385
Transcript length (bp)	1,480	1,270
Exon length (bp)	570	372
Intron length (bp)	84	56
Protein length (aa)	421	347
Exons per gene	2.60	2
**B**		
**Repeats and transposable elements**	**Number of elements**	**Length occupied**
SINEs	25	1,679 bp
MIRs	6	433 bp
LINEs	192	14,456 bp
LINE1	11	882 bp
LINE2	28	2,238 bp
L3/CR1	68	4,700 bp
LTR elements	2	106 bp
ERVL-MaLRs	1	64 bp
ERV_classII	1	42 bp
DNA elements	45	3,689 bp
hAT-Charlie	8	560 bp
Total interspersed repeats		19,930 bp
Small RNA	88	22,102 bp
Simple repeats	14,030	590,502 bp (1.32%)
Low complexity	1,929	98,961 bp (0.22%)

*Scaffold N50, scaffold length at which 50% of total bases in the assembly are in scaffolds of that length or greater. Scaffold L50, number of scaffolds whose sum length is N50*.

Transcript profiling was conducted in the context of a compatible interaction between the susceptible *H. brasiliensis* clone PB260 and *C. cassiicola* reference isolate CCP. Rubber trees were grown in a greenhouse at 28°C during the day (16 h) and 26°C at night (8 h), with 80% relative humidity. Leaves were sampled at physiological stage C according to Hallé and Martin (Hallé and Martin, 1968), and placed in large Petri dishes, on moist paper. Spore suspensions were obtained from 7-day-old mycelium cultures on PDA medium, washed with sterile water (4-5 ml per Petri dish), and filtered through a 100 μm cell strainer (Biologix). Inoculations were as follows: six 20 μl droplets of spore suspension at a concentration of 5,000 spores/ml were spotted on the abaxial face of the detached leaflets and incubated at 26°C, 60% relative humidity (Déon et al., 2012a). Two leaf discs (2.2 cm^2^) per leaflet were collected at the inoculation spots using a cork borer, 24 and 48 h after inoculation (from distinct leaflets), with three independent biological repeats for each time point. In addition, we used two independent suspensions of germinating spores (kept in water for 4 h after collection) as reference for differential gene expression analysis.

### Nucleic acid preparation and sequencing

Mycelium was flash frozen and ground in liquid nitrogen. For the reference genome, total DNA was extracted from 500 mg of ground material according to the 1,000 Fungal Genomes Project recommended protocol (http://1000.fungalgenomes.org/home/protocols/high-quality-genomic-dna-extraction/). In brief, the protocol consisted in a crude extraction using CTAB buffer followed by a purification step using Qiagen 5,000/G Genomic Tips (Qiagen, Courtaboeuf, France). Quality control was ensured by agarose gel migration and by NanoDrop1000 spectrophotometer quality assay (Thermo Fisher Scientific, Wilmington, U.S.A.).

The *C. cassiicola* CCP genome and transcriptome were sequenced using Illumina, the former in a combination of fragment and long mate pair (LMP) libraries.

For the fragment library, 100 ng of DNA was sheared to 300 bp using the Covaris LE220 and size-selected using SPRI beads (Beckman Coulter). The fragments were treated with end repair, A-tailing, and ligation of Illumina-compatible adapters (IDT, Inc.,) using the KAPA-Illumina library creation kit (KAPA biosystems).

LMP was produced from 6 μg of DNA sheared using the Covaris g-TUBE™ (Covaris) and gel size selected for 4 kb. The sheared DNA was treated with end repair and ligated with biotinylated adapters containing loxP. The adapter ligated DNA fragments were circularized via recombination by a Cre excision reaction (NEB). The circularized DNA templates were then randomly sheared using the Covaris LE220 (Covaris). The sheared fragments were treated with end repair and A-tailing using the KAPA-Illumina library creation kit (KAPA biosystems) followed by immobilization of mate pair fragments on strepavidin beads (Invitrogen). Illumina-compatible adapters (IDT, Inc) were ligated to the mate pair fragments and 10 cycles of PCR were used to enrich for the final library (KAPA Biosystems).

Stranded cDNA libraries were generated using the Illumina Truseq Stranded RNA LT kit. mRNA was purified from 1 μg of total RNA using magnetic beads containing poly-T oligos. mRNA was fragmented and reverse-transcribed using random hexamers and SSII (Invitrogen) followed by second strand synthesis. The fragmented cDNA was treated with end-pair, A-tailing, and adapter ligation, and 10 cycles of PCR were used to enrich for the final library.

The prepared library was quantified using KAPA Biosystem's next-generation sequencing library qPCR kit and run on a Roche LightCycler 480 real-time PCR instrument. The quantified library was then multiplexed with other libraries, and the pool of libraries was then prepared for sequencing on the Illumina HiSeq sequencing platform using a TruSeq paired-end cluster kit, v3, and Illumina's cBot instrument to generate a clustered flowcell for sequencing. Sequencing of the flowcell was performed on the Illumina HiSeq2000 sequencer using a TruSeq SBS sequencing kit, v3, following a 2 × 100 bp (LPM) or 2 × 150 bp (fragments and transcriptome) indexed run recipe.

For the other isolates, DNAseq library preparation (Illumina TruSeq v3, 2 × 100 bp) and sequencing was carried out at the GenoToul GeT Platform (INRA Auzeville, France) on the Illumina Hiseq2000 platform.

For the reference transcriptome (CCP mycelium grown in liquid culture), RNA was extracted from the flash-frozen mycelium as previously described (Chang et al., 1993) and sequenced by JGI using Illumina.

For RNA-Seq kinetic analysis, the collected material (leaf discs from inoculated leaves and germinating spores used as reference) was immediately flash-frozen. For the germinating spores, 60 Petri dishes were required to yield sufficient material. Spores were collected in water as described above and the suspension was maintained for 4 h at room temperature and concentrated by centrifugation (20 min at 6,000 *g*). The supernatant was discarded and the pellet was ground in liquid nitrogen. In both cases, total RNA was extracted as previously described (Chang et al., 1993). RNA-Seq library preparation (Illumina TruSeq v3, 2 × 150 bp) and sequencing was carried out at the GenoToul GeT Platform (INRA Auzeville, France) starting from 2 μg of total RNA, on the Illumina Hiseq2000 platform.

### *In silico* analyses

#### Genome *de novo* assembly

For the reference genome, Illumina genomic reads from two libraries were filtered and assembled with AllPathsLG (Gnerre et al., 2011). RNA-seq data for each genome were *de novo* assembled into consensus sequences using Rnnotator (Martin et al., 2010). The genome assembly was annotated using the JGI Annotation Pipeline and made available via the JGI fungal genome portal MycoCosm (jgi.doe.gov/fungi; Grigoriev et al., 2014). The Whole Genome Shotgun project of the reference isolate CCP has been deposited at DDBJ/ENA/GenBank under the accession NSJI00000000. The version described in this paper is version NSJI01000000.

For additional strains, after quality control, short reads were assembled in contigs using Velvet v.1.2.10 (Zerbino and Birney, 2008) with k-mers hash tables of 57 and automatic coverage cutoff. Mapping back the short reads to the generated contigs validated assembly parameters, yielding >97% mapped and >90% properly paired reads with the BWA SAMPE algorithm (Li and Durbin, 2009).

Post-assembly/annotation analysis relied on a parallel implementation (Tange, 2011) hosted by an Ubuntu 12.10 x64 server running on a 32-thread Intel Xeon Dell PowerEdge R720 server.

The Whole Genome Shotgun project of the additional *C. cassiicola* strains (BioProject PRJNA428435) has been deposited at DDBJ/ENA/GenBank under the accessions POQW00000000 to POSE00000000. The versions described in this paper are versions POQW01000000 to POSE01000000.

#### Functional annotation

Transposable elements (TEs) and repeats were searched for in the *C. cassiicola* reference genome assembly by first using RepeatScout v1.05 for *de novo* identification of simple repeats and then RepeatMasker open-4.0.5 (Smit et al., 2013–2015) with RepBase Update 20140131, RMLib 20140131 and Dfam v1.3 (Wheeler et al., 2013) and NCBI rmblastn v.2.2.27+ (Altschul et al., 1990; Camacho et al., 2009).

Carbohydrate-active enzymes (CAZymes) were predicted in fungal genomes using predicted protein sequences as queries and CAZyme Analysis Toolkit (Park et al., 2010) and the CAZy database (Lombard et al., 2014) (http://www.cazy.org). Proteins were considered as putative CAZymes if detected by both the Blast (*E* = 1e-5) and Pfam methods.

Lipases were identified in fungal genomes using predicted protein sequences as queries and hmm profiles from the Lipase Engineering Database v3 (http://www.led.uni-stuttgart.de) and hmmsearch (HMMER 3.1b1; http://hmmer.org/) with an *E*-value inclusion threshold set at 0.01.

Proteases were identified in fungal genomes using predicted protein sequences as queries and the MEROPS v9.12 blast database (Rawlings et al., 2014) (http://merops.sanger.ac.uk/) with Blastp (*E* = 1e-4).

Genes putatively involved in the secondary metabolism were searched for: non-ribosomal peptide synthases (NRPSs), polyketide synthases (PKSs) and terpene synthases (TSs) were identified using predicted protein sequences as queries and a local distribution of antiSMASH v2.0 (Blin et al., 2013).

Proteins were predicted as potentially secreted when the following two conditions were satisfied: (i) absence of a transmembrane domain identified by our TMHMM v2.0c local server (Sonnhammer et al., 1998), and (ii) presence of a secretion peptide signal as predicted by SignalP v4.1 with the Eukaryote database (Petersen et al., 2011). Proteins potentially secreted through ER/Golgi-independent pathways were not taken into account in this study.

#### Protein clustering

Clusters of homologous proteins were computed using the latest available standalone version of OrthoMCL 2.0.9 (Li et al., 2003; Fischer et al., 2011). Protein sequences were first filtered using the default complexity setting (orthomclFilterFasta <PATH> 10 20) yielding 578,863 proteins concatenated into a single blast database. All-versus-all blastp was then carried out with NCBI Blastp v2.2.30+ with the *E*-value filter set at 1e-5. MCL (Enright et al., 2002) was then used with the inflation parameter set at 1.5. We determined this value experimentally using a manually curated cluster of orthologous major intrinsic proteins (MIPs), X-intrinsic proteins (XIPs). Using inflation set at 1.5, we obtained the least number of protein clusters while keeping XIPs distinct from other MIP families. Cluster dynamics was assessed using CAFE v3.1 (Han et al., 2013).

#### Phylogenies

To build an interspecific phylogeny of the 45 fungal species of this study, we used predicted protein sequences found in exactly one copy in all fungal representatives, on the basis of cluster analysis. These 651 protein sequences of each fungal representative were individually aligned using MAFFT v7.215 (Katoh and Standley, 2013) with the default algorithm. Informative residues were extracted using Gblocks 0.91b (Castresana, 2000) using default settings considered by the author as stringent (minimum number of sequences for a conserved position 9; minimum number of sequences for a flank position 14; maximum number of contiguous non-conserved positions 8; minimum length of a block 10; gaps not allowed). After trimming, individual protein alignments were tested to obtain the best evolutionary model according to AIC scores using ProtTest v3.4 (Guindon and Gascuel, 2003; Darriba et al., 2011). We found that the LG model was the best substitution matrix with our data, and so it was selected for maximum likelihood tree reconstruction using RAxML v8.1.16 (Stamatakis, 2014) with 1,000 bootstraps on the concatenated chimeric protein sequence. Interspecific phylogeny was computed on 120,063 residues for each of the 45 species.

For intraspecific phylogeny of the 37 *C. cassiicola* isolates, the core set of 651 protein sequences previously used for the interspecific phylogeny was insufficient to discriminate closely related isolates (ie. the chimeric sequences were identical between CCAM1 and CCAM4, between IA and JQ, between CSRI1, CSRi2, and TSB1, and between CTHA1, CTHA3, and CTHA4). We thus used a larger set of 12,112 protein sequences found in exactly one copy in each of the 37 *C. cassiicola* isolates studied. After Gblocks selection, only the polymorphic residues were conserved in the alignment using SNP-sites (Page et al., 2016) yielding 37 chimeric concatenated sequences of 300,213 residues for final maximum likelihood tree calculation using RAxML with LG substitution matrix and 1,000 bootstraps. Tree rendering was carried out using FigTree v1.42 (http://tree.bio.ed.ac.uk/software/figtree/).

#### Statistical analysis

Data management and statistical analysis were carried out using R version 3.2.1 (R Core Team, 2015) (http://www.R-project.org/). PCA analyses used FactoMineR package (Lê et al., 2008). RNA-Seq expression was rendered using the heatmap.2 function of the plots package (Warnes et al., 2016). Hierarchical clustering was computed using the R base package using Euclidean distances and the Ward method. Gene ontology enrichments were computed by a chi-squared test between observed and expected abundances and *p*-values were adjusted with a 0.01 FDR under a dependent multiple hypothesis (GO abundances are not independent since they are graph-related) (Benjamini and Yekutieli, 2001).

#### RNA-seq analysis

RNA-Seq analysis was carried out using our custom pipeline (Garavillon-Tournayre et al., 2015) running on the GenoToul computing cluster. Briefly, 125-bp pair-end shorts reads were first decontaminated by mapping against a database of potential contaminants, then finally mapped on annotated CCP transcripts using the Burrows-Wheeler alignment algorithm BWA-MEM (Li and Durbin, 2009). Owing to the very small proportion of short reads that targeted fungal RNA (0.05%), counts for most genes were very low. Transcripts with fewer than three counts in at least two of the eight biological replicates (25%) were filtered out, allowing 1,052 transcripts for differential analysis. Differential expression analysis was carried out using the DEseq2 package (Love et al., 2014) with 0.01 FDR yielding 353 genes.

## Results and discussion

**Figure 1:**
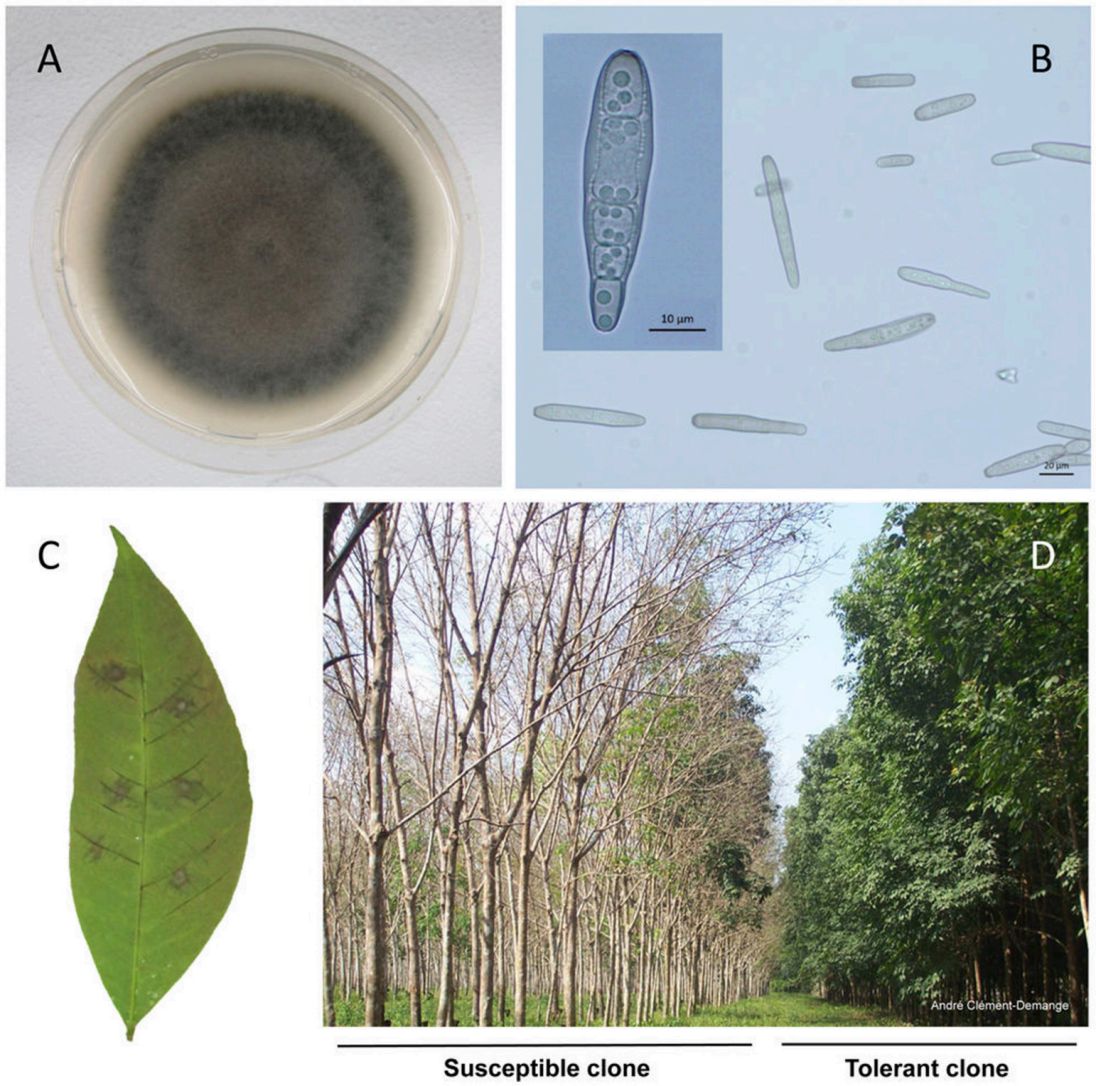
*Corynespora cassiicola* strain CCP and the *Corynespora* Leaf Fall (CLF) disease symptoms. **(A)** CCP mycelium colony on PDA medium 7 days after subculture. **(B)** Optical microscopy view of CCP conidia in water. **(C)** *Hevea brasiliensis* clone PB217 leaflet inoculated with CCP spores (7 days post-inoculation, 500 spores per spot). **(D)** CLF symptoms on a susceptible rubber tree clone (left, RRIC103) next to a tolerant clone (right, GT1), in Nigeria.

CCP was selected as the reference among a collection of *C. cassiicola* isolates from various hosts and of diverse geographical origins, for its high virulence in the rubber tree (Breton et al., 2000; Déon et al., 2012a,b, 2014). CCP mycelium grown on PDA was fluffy and whitish gray when young, turning dark gray when older (Figures 1A,B). The conidia varied in size (from 10 to 145 μm) and shape (from aseptate to six septates) (Figure 1C). Such variability is a known feature of the species (Qi et al., 2011). Conidia inoculation of detached leaves from sensitive rubber clones induces the so-called fishbone necrosis with typical darkening of the veins within 48 h (Figure 1D).

Sequencing of the *C. cassiicola* (alias Corc) CCP genome was performed by JGI as part of the 1000 Fungal Genomes Project using Illumina sequencing. The assembled genome covers 44.85 Mbp in 500 contigs, with an average depth of 58x (Table 1). Among the 17,167 predicted gene models, 12,261 (71%) have at least one type of annotation, whether Interpro domain (56.8% of the genes), Gene Ontology (46%), Kyoto Encyclopedia of Genes and Genomes (18.1%), or EuKaryotic Orthologous Groups (13.6%) annotations.

The relatively compact genome has small amounts of repetitive DNA and transposable elements. The 44.85 Mbp CCP genome is larger than the average of 36.91 Mb reported for Ascomycota but close to that of the Dothideomycetes (44.59 Mb) (Mohanta and Bae, 2015). Furthermore, the CCP genome has 17,167 gene models, which is very high compared with the ~11,000 found on average in Ascomycota and the ~13,000 in Dothideomycetes (Mohanta and Bae, 2015). Recently, the genome of *C. cassiicola* strain UM591, isolated from the contact lens of a patient diagnosed with keratomycosis in Malaysia, was published (Looi et al., 2017). The authors used both long and short insert sequencing libraries, allowing the assembly of 41.88 Mb with 1,941 contigs. The authors identified 13,531 protein coding genes in UM591, significantly fewer than the 17,167 identified by the DOE-JGI annotation pipeline. Taken together, both analyses suggest that the *C. cassiicola* genome has undergone expansion.

### PART 1. interspecific comparative genomics

In the first part of this study, we present an interspecific comparative analysis of the CCP predicted proteome along with 44 species sequenced by the DOE-JGI (Table S1), representatives of diverse fungal lifestyles (i.e., saprotrophic, necrotrophic, hemibiotrophic, biotrophic, ectomycorrhizal). The selected species included six Basidiomycota and 38 Ascomycota. Special emphasis was placed on the Dothideomycetes class (23 species), which comprises many plant pathogens, including *C. cassiicola*. Altogether, 31 genera from 10 classes were represented in this study, thus maximizing the diversity of gene molecular functions.

### Interspecific genome-wide phylogeny

**Figure 2:**
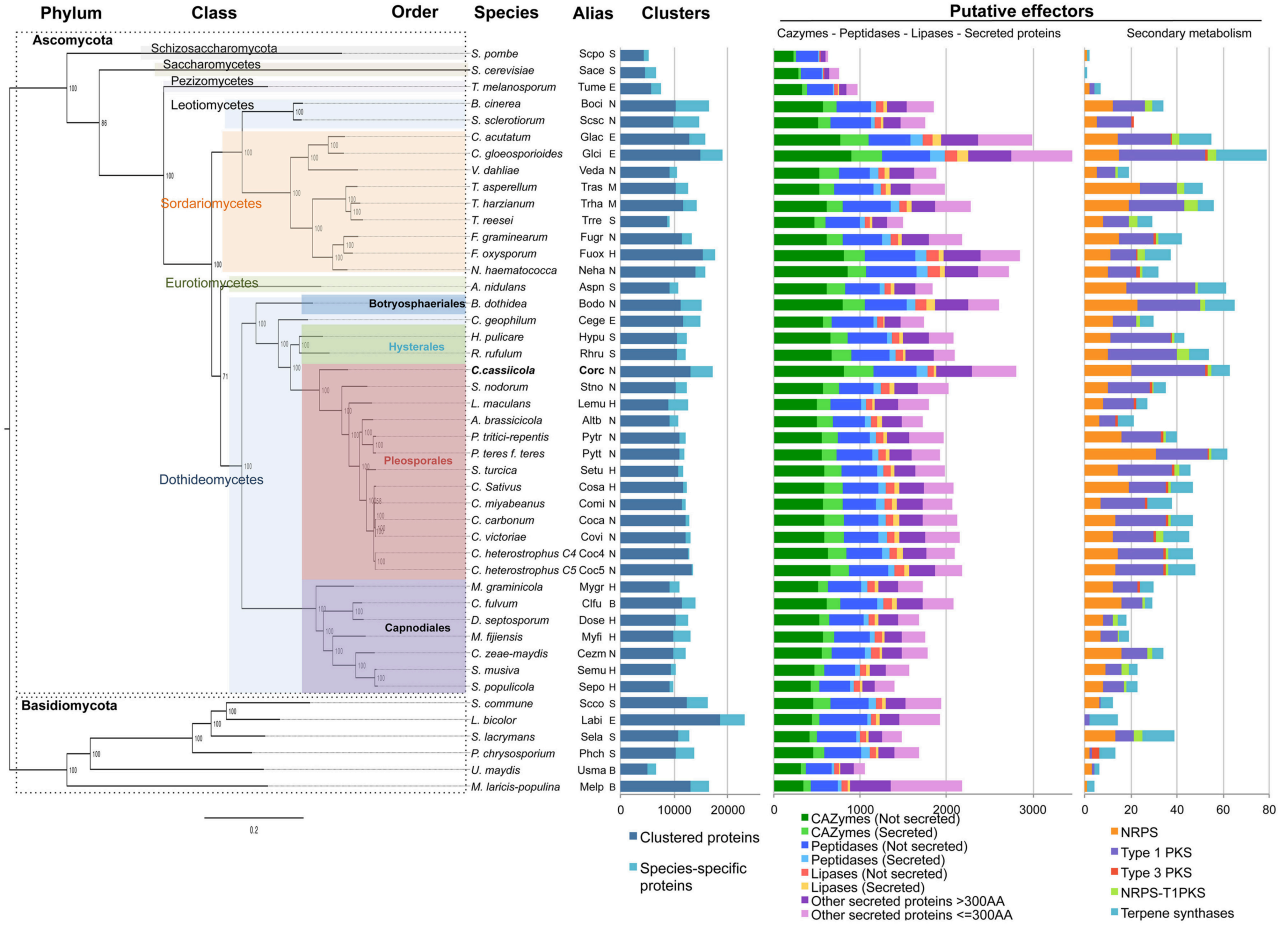
Interspecific phylogenomic tree, protein clusters and putative effectors of 45 fungal species. Details of the species and their genome access links are listed in Table S1. Life styles are indicated by a letter: S, saprotrophic; N, necrotrophic; H, hemibiotrophic; B, biotrophic; E, ectomycorrhizal. The maximum likelihood phylogenetic tree was based on 651 concatenated core protein sequences. Branch lengths are indicated by the bar (substitutions/site); 1,000 bootstrap values are shown as percentages. Clustered and non-clustered (species-specific) protein numbers were predicted based on OrthoMCL clustering. Putative effectors: number of gene models encoding CAZymes, peptidases, lipases and other secreted proteins (left diagram), or involved in the secondary-metabolism (NRPS, PKS, terpenes synthases, right diagram).

To evaluate the dynamics of the *C. cassiicola* gene families, and more especially of those involved in virulence (*i.e*. effectors), we clustered the 578,823 predicted protein sequences identified in the 45 fungal genomes studied here. All-versus-all blastp analysis gave 68 million hits with *E* > 1e-5. We used this input to carry out a Markov clustering (MCL algorithm). Since there is a trade-off between sensitivity and specificity, we experimentally tuned clustering parameters to yield the most meaningful clusters using a manually expertized gene family, the X-intrinsic proteins (XIPs), a subfamily of the large major intrinsic proteins (MIP) family (Lopez et al., 2012). Clustering parameters were tuned to obtain the fewest protein clusters while keeping XIPs distinct from other MIP families. As a result, 480,841 proteins predicted from the 45 genomes fell into 40,701 clusters, composed of a maximum of 508 genes with a mean of 11.81 genes and a median of 4 genes per cluster. The CCP genome has a high percentage of singletons (23.2%) compared with the other species (16.5% on average across all 45 species, and 10.5% across the 13 Pleosporales), the highest (37.9%) being found in *Botrytis cinerea* (Figure 2). As expected, few CCP singletons (461 only) have predicted GO functional annotations (Tables S2, S3).

We built a phylogenomic tree of the 45 fungal species based on proteins sequences having exactly one representative in each species. With our parameters, 651 clusters met with this criterion. These core sequences were concatenated into a chimeric sequence used for phylogenetic distance calculation and tree construction (Figure 2). The computed output is in agreement with the Fungal Tree of Life (Celio et al., 2006; Schoch et al., 2009) and places CCP in a distinct clade among the Pleosporales. Our genome-wide phylogeny confirms with very high support that *C. cassiicola* speciation has occurred before the speciation of the Pleosporinae suborder, which includes several families of plant pathogens such as the Phaeosphaeriaceae, the Laeptosphaeriaceae and the Pleosporaceae.

### CCP putative effectors

In this study, effectors are broadly defined as fungal proteins that can modulate plant immunity. Two main classes of effectors were distinguished (Figure 2). One includes enzymes interacting with plant substrates (CAZymes, peptidases and lipases), together with secreted protein of still unknown function, with a distinction between SSPs (“small secreted proteins” of 300 amino acids or fewer) and LSPs (“large secreted proteins”). Only classically-predicted secreted proteins (i.e., proteins with a signal peptide but no transmembrane domain) were considered in this study, but we cannot rule out the possibility that non-canonical proteins may also act as effectors. The second class of effectors corresponds to enzymes associated with the secondary metabolites. To allow comparative genomics analysis, we predicted the putative effectors simultaneously for *C. cassiicola* and the other 44 species. In terms of quantity, *C. cassiicola* appeared to be particularly rich in putative effectors compared with the other species. A detailed list of CCP putative effectors is given (Table S4) and discussed below.

#### CAZymes

CAZymes play a substantial role in carbon acquisition and metabolism, and thus in the heterotrophic lifestyle of fungi (Zhao et al., 2014). They have been grouped into six classes (Lombard et al., 2014): glycoside hydrolases (GHs), glycosyl transferases (GTs), polysaccharide lyases (PLs), carbohydrate esterases (CEs), auxiliary activities (AAs), and carbohydrate-binding modules (CBM). In pathogens, GTs are said to mainly ensure carbohydrate assembly, while CEs, GHs, and PLs are cell wall degrading enzymes (CWDEs).

In CCP, we found a total of 1,152 proteins sharing homology with CAZymes (Figure 2, Table S4). Among these, 326 (29%) have a secretion signal peptide, including 47 SSPs and 279 LSPs. Among the 45 species of our study, CCP ranked second richest in CAZymes after the hemibiotroph *Colletotrichum gloesporioides* (Glci). CCP predicted CAZyome comprised 417 putative GHs, 305 GTs, 209 AAs, 148 CEs, 42 CBMs, and 31 PLs. Of the predominant family, the GHs, 40% belonged to five classes: GH92 (52), GH76 (40), GH3 (28), GH43 (27), GH18 (23). GH92s are exo-acting α-mannosidases and GH76s are endo-acting α-mannanases. Mannans being among the most abundant components of hemicellulose, the mannan endo- and exohydrolysing enzymes could together play a role in softening host tissues (Moreira and Filho, 2008; Rodríguez-Gacio Mdel et al., 2012).

The most represented CAZyme modules were GT34 (121), CE10 (91), AA7 (64), AA3 (56), GH92 (52), GT2 (44), and AA9 (37). GT34s ensure hemicellulose degradation. CE10s may be involved in fungal pathogenesis (Islam et al., 2012; Aragona et al., 2014; Zhao et al., 2014; Kuan et al., 2015), although most of them have been described as acting on non-carbohydrate substrates (Cantarel et al., 2009). AA7s and AA3s have catalytic modules involved in enhancing plant cell wall degradation (Levasseur et al., 2013); GH92s are mannosidases; GT2s ensure chitin synthesis; AA9s are believed to act directly on cellulose, working in concert with AA3s for oxidative cleavage, but some of them also degrade chitin and starch (Quinlan et al., 2011; Levasseur et al., 2013). In addition, Corc has 58 pectin degrading enzymes (14 PL1s, 14 PL3s, 17 GH28s, and 9 GH78s) that could also participate in host cell wall degradation. Finally, in an extensive survey of CAZyme contents conducted on 103 diverse fungi (Zhao et al., 2014), the authors noted the expansion of PL1 and PL3 in necrotrophs.

The high CAZyme content of CCP is consistent with its life style as a necrotrophic pathogen.

#### Lipases

Lipases form an emerging field of research into virulence mechanisms in fungi. Lipases (triacylglycerol acylhydrolases E.C. 3.1.1.3) hydrolyse carboxyl ester bonds on triacylglycerols, and belong to the class of α/β hydrolase fold enzymes. Notably, secreted lipases may act against host morpho-anatomical barriers (wax and cuticle). We identified 104 putative lipases in CCP, including 40 with secretion signals (27 SSPs and 13 LSPs) (Figure 2, Table S4). Compared with the other species, with a mean value of 127 lipases, CCP had one of the lowest numbers of lipase representatives, the maximum being observed for Glci with 271 lipases.

Cutinases (E.C. 3.1.1.74) are esterases that hydrolyse cutin polymers into cutin monomers. They can be annotated as members of the CE5 class of CAZymes or as lipases, since they also belong to the α/β hydrolase fold class of enzymes, and are reported as the abH36 superfamily in the Lipase Engineering Database (http://www.led.uni-stuttgart.de). As leaf cuticles are partly composed of cutin, cutinases are thought to participate in pathogenesis through host recognition, spore adhesion, enhancing mycelium, growth, appressorium differentiation, pathogen penetration, and carbon acquisition (van Kan et al., 1997; Comménil et al., 1998; Davies et al., 2000; Reis et al., 2005; Skamnioti and Gurr, 2009; Liu et al., 2016). Globally, necrotrophs have more cutinases than hemibiotrophs while saprotrophs and ectomycorrhizal fungi tend to have few to none. Our analysis again ranks CCP among the best equipped species, with 11 putative cutinases (Table S4).

#### Peptidases

Peptidases, also known as proteinases, proteases and proteolytic enzymes, are responsible for protein degradation (proteolysis) and also belong to the α/β hydrolase superfamily. In fungal pathogens, secreted peptidases could serve for both nutrition and the degradation of host defense proteins, and could thus interfere with plant innate immunity (Carlile et al., 2000; Olivieri et al., 2002; Rodríguez-Herva et al., 2012). The role of peptidases in pathogen virulence is supported by the upregulation of peptidase inhibitors in *Fusarium* head blight resistant cultivars (Gottwald et al., 2012). Peptidases are considered potential targets for disease control using peptidase inhibitors (Lowe et al., 2015). We identified 630 peptidases in the CCP genome, of which 124 harbored a secretion signal (Figure 2, Table S4). Here again, CCP ranked as one of the richest for this class of effectors after *F. oxysporum* (Fuox), *N. haematococca* (Neha) and the two *Colletotrichum* species (Glci and Glac). Overall, serine-, metallo- and cysteine-peptidase families dominate CCP peptidase content, with respectively 328, 173, and 88 representatives. For Ohm et al. (2012), Dothideomycetes have more serine-peptidases S10 and metallo-peptidases M14, but fewer aspartic peptidases (A01) than other plant pathogenic necrotrophs. In the case of CCP, these families were close to the average over all necrotrophs (12 *vs*. 11 for S10, 19 *vs*. 21 for A01, and 7 *vs*. 6 for M14).

#### Other secreted proteins

The CCP genome encodes 1,411 proteins with a predicted secretion signal, which represents 8% of the total proteome. Of these secreted proteins, only 35% fall into one of the effector categories described above (326 CAZymes, 124 peptidases and 40 lipases, Figure 2). The others comprise 411 LSPs and 510 SSPs: 50% of all LSPs and 85% of all SSPs (Table S4). We carried out a GO enrichment analysis on the “other secreted proteins” subcategory (Table S5) and found an over representation of hydrolase activities (GO:0016787, GO:0016788), related to carbohydrates (GO:0005975), and also lipid metabolism (GO:0006629). Six genes annotated with serine-type peptidase activity (GO:0008236) contribute to the overrepresentation of the term (five of the subfamily S41 and one S26). However, these genes were not included in the peptidase category, since they did not meet the threshold E-value in our analysis. Redox processes largely contribute to the overrepresented GO terms of the “other secreted proteins” category. Among them, seven oxidases with FAD binding (GO:0050660) annotation were found including two members with berberine/berberine-like domains thought to play a role in ROS production. The electron transport biological process (GO:0005506) was also found significantly overrepresented, notably due to the contribution of seven heme-binding proteins (GO:0020037) among which four cytochrome P450, one cytochrome B5, one peroxidase and one catalase.

As expected, very few “other SSPs” had annotations compared with “other LSPs.” In *C. cassiicola*, the only SSP characterized to date as an effector is the cassiicolin toxin (Barthe et al., 2007; de Lamotte et al., 2007) encoded by *Cas1* in CCP (Déon et al., 2012a). This special case will be addressed in Part4.

#### Secondary metabolism genes

Genes involved in the secondary metabolism play an important role in fungal pathogenic lifestyle (Ohm et al., 2012). Polyketide synthases (PKSs), non-ribosomal peptide synthetases (NRPSs) and terpene synthases are involved in the production of polyketide, peptides and terpenes respectively. Our predictions revealed that the CCP genome encodes 20 NRPS, 32 type-1 polyketide synthases (T1PKS), 1 type-3 PKS (T3PKS), 2 hybrid NRPS-PKS proteins and 8 terpenes synthases (Figure 2, Table S4). With a total of 63 genes related to the secondary metabolism, against 34 on average, CCP ranked fourth among all 45 species, second among Dothideomycetes, and first among Pleosporales. Several of these genes were found physically clustered in the genome (Table S4).

#### Comparison of CCP and UM591 putative effectors

We compared our results with those recently published by Looi et al describing the *in silico* identification of putative effectors in the human-hosted *C. cassiicola* isolate UM591 (Looi et al., 2017). Both studies (UM591 *vs*. CCP) yielded similar quantities of predicted lipases (105 *vs*. 104), cutinases (9 *vs*. 11) and secondary metabolism genes (50 *vs*. 63), but differed in peptidases (216 *vs*. 630) and CAZymes (973 *vs*. 1152). Notably, the most strongly represented CAZyme module in CCP (GT34, 121 members) was poorly represented in UM591 with seven members only. Conversely, the xylan esterases CE1s were among the most represented in UM591 (49) while CCP had only five. For the other CAZymes, counts were in similar ranges in both isolates. We are nonetheless confident in our counts because our annotation pipeline yielded results similar to those described for the other plant-hosted fungi studied here, as described in Part 1. Furthermore, numerous effector subfamily counts were similar between UM591 and CCP. Although annotation differences between the two studies cannot be ruled out, the hypothesis that UM591 gene loss for some but not all CAZymes families and peptidases could reflect a specialization toward non-plant hosts deserves further examination.

### Interspecific comparison based on all putative effectors

**Figure 3:**
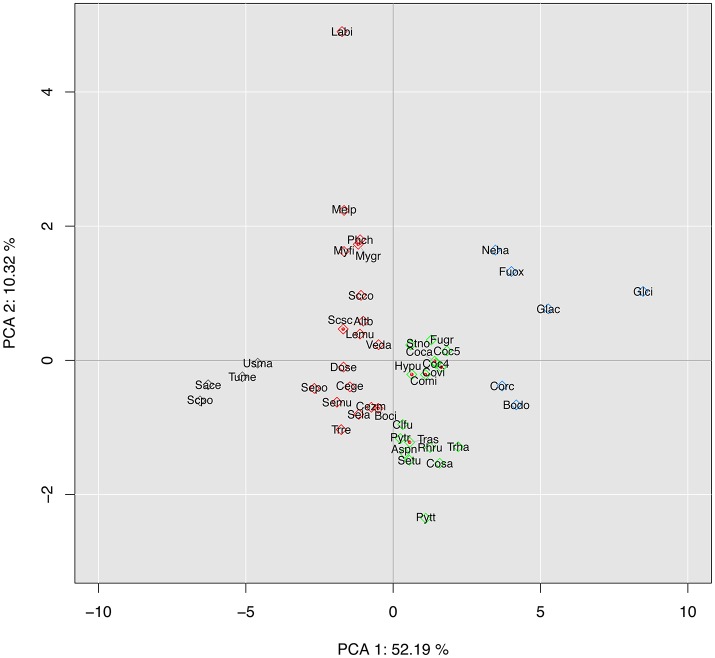
Interspecific principal component analysis (PCA) of 45 fungal species based on their putative effector composition. Species are represented by their alias name (Table S1). PCA was computed from the counts in each effector category as represented in Figure 2 (14 dimensions). Different colors represent different clusters.

We carried out a principal component analysis (PCA), based on the whole repertoire of putative effectors, for the 45 plant-associated species of our study (Figure 3). This analysis identified four main clusters, with no clear consistency for phylogeny or lifestyle (Figure 2 and Table S1).

CCP is the only Pleosporales member (1/24) in a cluster grouping the only Botryosphaeriales representative, *Botryosphaeria dothidea* (Bodo), and four Sordarimycetes (4/9), namely the two *Colletotrichum* species *C. gloesporioides* (Glci) and *C acutatum* (Glac), *Nectria haematococca* (Nema), and *Fusarium oxysporum* (Fuox) (Figure 3, blue cluster). These six species are described as necrotrophic or hemibiotrophic. They are characterized as having a high number of total genes and the highest number of putative effector genes in the CAZyme, peptidase, lipase and other secreted protein categories (Figure 2).

Most (10/13) of the other Pleosporales members form a cluster (green) with seven other Ascomycetes belonging to three classes (Sordariomycetes, Eurotiomycete and Dothideomycetes). Most lifestyles are represented in this cluster: *Trichoderma asperellum* (Tras) and *T. harzianum* (Trha) are mycoparasitic; *Aspergilus nidulans* (Aspn), *Hysterium pulicare* (Hypu) and *Rhytidhysteron rufulum* (Rhru) are saprophytic, and all the others are pathogenic (biotrophic, necrotrophic or hemibiotrophic).

The last two Pleosporales species, *Leptosphaeria.maculans* (Lemu) and *Alternaria brassiicola* (Altb), stand in a phylogenetically highly diverse cluster (red), including both Basidiomycetes (5/6) and Ascomycetes belonging to five classes. Here again, most lifestyles are represented, with both pathogenic and non-pathogenic species.

The last cluster (black) is composed of the four species with the lowest numbers of total and effector genes in this selection (Figure 2), i.e., the Ascomycetes *Saccharomyces pombe* (Scpo), *Saccharomyces cerevisiae* (Sace), and *Tuber melanosporum* (Tume), together with the Basidiomycetes *Ustilago maydis* (Usma). While the three Ascomycetes are non-pathogenic (saprotrophic or ectomycorrhizal), *U. maydis* is a plant pathogen of maize and teosinte, emphasizing that even species with a small genome and a small effector arsenal can be pathogenic, though with a narrow host range (Kämper et al., 2006).

Of the 15 components of this PCA analysis, the first two dimensions covered over 62% of the total variance of the dataset. The first dimension, which accounts for 52% of the total variance, shows a large contribution from CAZymes and a smaller one from lipases and peptidases, while the second dimension shows the contribution of secondary metabolism genes. When PCA was based on CAZymes only (Figure S1), CCP was grouped with the six pathogenic species of the Sordariomycetes order, but remained separated from the other Pleosporales; *B. dothidea*, previously grouped with CPP, formed a single-species cluster. When PCA was based on the content of genes associated with the secondary metabolism (Figure S2), CCP was found closely associated with *P. teres* f. sp. *teres* (Pytt), another necrotrophic species belonging to the Pleosporales, although the overall clustering could be related neither to phylogeny nor life style.

In conclusion, *C. cassiicola* isolate CCP was consistently associated with *C. acutatum* (Glac) and *F. oxysporum* (Fuox) whatever the effector categories, and to a lesser extent with *C. gloesporioides* (Glci), *N. haematococca* (Neha) and *B. dothidea* (Bodo). All these genomes have in common expansions in most categories of effectors. This clustering agrees weakly with phylogeny but seems more relevant to lifestyle considerations such as pathogenicity and nutrient uptake mechanisms: all six species are known to have broad host ranges and potentially multiple trophic modes.

*Colletotrichum* is a widespread genus of some 600 species causing necrotic spots and rot in a wide range of plants. It is a hemibiotrophic fungus able to operate lifestyle transitions during its interaction with the host plant, switching from epiphytic growth to biotrophy and finally to necrotrophy. The duration of the symptomless phase may vary significantly between species. Some *Colletotrichum* species have been clearly described as non-pathogenic plant endophytes conferring fitness benefits to their host, while others have been described as saprophytes ensuring survival on soil debris (Cannon et al., 2012; O'Connell et al., 2012; Hiruma et al., 2016). *C. gloesporioides* and *C. acutatum* species have been described as pathogens of the rubber tree (Brown and Soepena, 1994), although species of the *C. gloesporioides* complex have also been isolated from asymptomatic rubber tree (Gazis et al., 2011). Interestingly, *C. cassiicola* and *Colletotrichum* spp. are frequently co-isolated from diseased rubber leaves (unpublished result) indicating that both are adapted to the same host and cellular environment. Their proximity in terms of putative effector contents is consistent with this observation.

*F. oxysporum* and *N. haematococca* (teleomorph of *F. solani*) are also cosmopolite species able to colonize a wide variety of environments, as saprobes or necrotrophic pathogens (Fravel et al., 2003; Coleman et al., 2009). *B. dothidea* is also a widespread latent pathogen, virtually present as an endophyte in all examined woody plants, and switching to pathogenicity under stress conditions (Slippers and Wingfield, 2007). However, none of these species has yet been found in the rubber tree, although species of the same genus have occasionally been described (Gazis and Chaverri, 2010). Most fungi species so far associated with rubber were isolated after surface-sterilization of the explant. A number of species with an epiphytic phase may thus have been dismissed. A recent study emphasized the role of epiphytic growth prior to invasion in the pathogenic fungal species *Zymoseptoria tritici* (Fones et al., 2017), and the bias it may create for interpreting the life style and infection strategies of the pathogen.

*Corynespora cassiicola* fits with this model of polymorph species with a broad host range. The CCP isolate is known to be necrotrophic and highly virulent in the rubber tree, but the conditions required for disease onset remain unclear and symptom intensities vary according to the cultivar. The fungus may survive as an endophyte or latent pathogen in living tissues, or as a saprobe on decaying material, and may potentially switch to necrotrophy under favorable conditions. Very numerous and diverse hosts of *C. cassiicola* have been described, including some outside the plant kingdom, with animal- and human-associated isolates. Nevertheless, individual *C. cassiicola* isolates display some host specialization, being restricted to a limited host range (Barthe et al., 2007; Dixon et al., 2009). Large effector repertoires may confer a greater chance of manipulating the defenses and escaping immunity in a wider range of hosts. By contrast, pathogens adapted to a narrow range of hosts maintain only a reduced set of effectors (O'Connell et al., 2012).

### PART 2. transcriptome analysis of CCP compatible interaction with rubber tree

As presented above, CCP contains 2,870 genes encoding putative effectors. To identify the candidates most likely to be involved in virulence in the rubber tree, we monitored the CCP transcriptome using RNA-Seq during the compatible interaction between CCP and the susceptible rubber tree clone PB260. Leaflets were inoculated with droplets of spore suspension. Germinating spores were used as reference for the differential analysis. Leaf disk samples were harvested 24 and 48 h post-inoculation. This early time frame was chosen to cover the initial stages of successful colonization: spore germination, mycelium penetration and development in the plant tissues, until the onset of the first necrosis symptoms. These symptoms are preceded by transient activation of the cassiicolin-encoding gene (Déon et al., 2012a,b).

In the germinating spores, we found that most of the 17,167 genes were expressed (Table 2, Table S6); only 2,179 transcripts had no sequencing reads coverage at all. However, the transcriptome mean coverage per gene was 457.7 while the median was only 49.5. This indicates that most CCP genes, though expressed, had low transcriptional activity in the context of spore germination outside a host.

**Table 2:** Metrics of the CCP gene transcripts.

Number of transcripts expressed in germinating spores	14,988
Differentially expressed in inoculated rubber tree leaves	353
Putative effectors	92
Others	261
Non differentially expressed in inoculated rubber tree leaves	14,635
Putative effectors	2,397
Others	12,238
Number of transcripts non expressed in germinating spores	2,179
Differentially expressed in inoculated rubber tree leaves	0
Non differentially expressed in inoculated rubber tree leaves	2,179
Putative effectors	381
Others	1,798
Total	17,169

**Figure 4:**
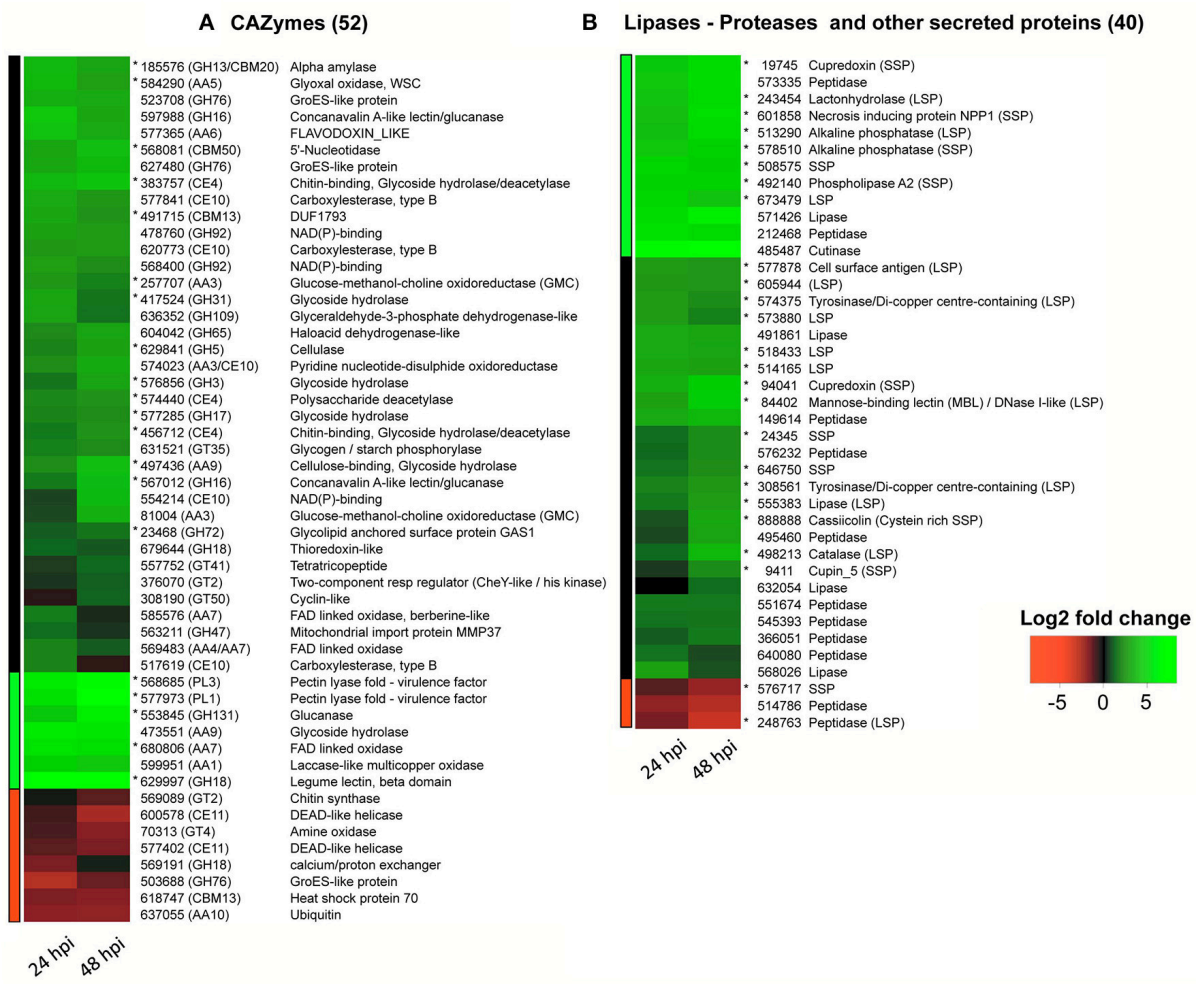
RNA-Seq heatmaps of CCP putative effectors differentially expressed 24 and 48 h after spore inoculation on detached rubber tree leaves (susceptible clone PB260). Differential expression was calculated as the Log2 fold change at each time point using the spore suspension as reference. The code numbers refer to JGI transcript IDs (http://genome.jgi.doe.gov/Corca1) except for cassiicolin Cas1 (888888) which was manually annotated. Genes with predicted secretion signals are noted with an asterisk. Colored bars on the left side define the three clusters obtained by hierarchical classification of the differential expression values. **(A)** CAZymes; **(B)** other effectors.

In the inoculated leaf samples, only a tiny portion of the sequencing reads (0.05%) were mapped to the CCP transcriptome (Table 2), owing to the low fungal/plant cell ratio at this early stage of infection. Consequently, only the most differentially expressed genes were detectable. As a result, we found that 353 genes had their expression significantly modified during the early stages of infection (Table 2 and Table S6), among which 92 (26%) were categorized as putative effectors (Figure 4). More precisely, 52 were annotated with CAZyme modules and 40 categorized as lipases/phospholipases, peptidases or other secreted proteins. No gene of the secondary metabolism was found differentially expressed in this context.

### Differentially expressed cazymes (Figure 4A)

The 52 differentially expressed genes with CAZyme modules included 20 GHs, 11 AAs, 10 CEs, 6 GTs, 3 CBMs, and 2 PLs (respectively 4.7, 5.2, 6.7, 1.9, 7.1, and 6.5% of the total count in each category). Interestingly, fewer GTs (only 1.9%), responsible for carbohydrate assembly, were differentially expressed compared with CEs, GHs and PLs, described as cell wall degrading enzymes (CWDEs). Most of the differentially expressed CAZyme genes (84%) were upregulated. Twenty differentially expressed CAZyme genes (40%) had a predicted secretion signal (they are marked with an asterisk). All of them were up-regulated, and notably five with a very high fold change (between 7.1 and 9.6): the two putative pectin lyases (PL1 and PL3), one glucanase, one FAD-linked oxidase and one lectin beta domain-containing protein. They can be considered as good effector candidates potentially involved in cell wall degradation (Kubicek et al., 2014; Ismail and Able, 2016). Other up-regulated candidate effectors were potential actors of cell wall degradation: a secreted cellulase, two GH16s, four non-secreted CE10 carbohydrate esterases„ two AA9s cellulose-binding glycoside hydrolases, and two AA3s glucose-methanol-choline (GMC) oxidoreductases. Two upregulated CE4s with secretion signals annotated as “chitin-binding, glycoside hydrolase-deacetylase” could play a role in the camouflage of chitin oligomers that would normally be acting as elicitors in the host plant, as previously hypothesized (Tsigos and Bouriotis, 1995). A secreted alpha-amylase (185,576), along with 417,524, a secreted GH31 with predicted glycoside hydrolase activity, could ensure carbon acquisition. Finally, other CAZyme activities such as glycosylation (GTs) could be associated with post-translational modification of proteins delivered to the host that could modulate pathogen virulence (Kubicek et al., 2014). Notably, in CCP, the effector cassiicolin was shown to be glycosylated (Barthe et al., 2007).

None of the eight repressed CAZYme candidates had a predicted secretion signal. We can however underline the repression of a chitin synthase gene, potentially as part of a strategy to avoid chitin-induced elicitation of the plant defenses.

### Differentially expressed lipases, peptidases and other secreted proteins (Figure 4B)

Besides CAZymes, we found that 11 peptidases, seven lipases and 22 other secreted proteins were differentially expressed, representing respectively 1.7, 6.7, and 2.4% of the total count in each effector category. They were mostly up-regulated.

Of the 11 peptidases, nine were found to be upregulated, but none had a secretion signal, in contrast with previous studies on *F. graminearum* showing an enrichment in secreted peptidases among the upregulated gene clusters (Lowe et al., 2015). Two peptidases were down-regulated, including one potentially secreted.

Among the lipases, one was annotated as cutinase and another as phospholipase. Both genes were strongly upregulated, but only phospholipase was predicted to be secreted. The other lipases had no secretion signal, except for one (555,383).

The other secreted proteins comprised 14 LSPs and 11 SSPs. All were upregulated except for one down-regulated SSP. One of the upregulated SSPs is a necrosis-inducing peptide 1 homolog (NPP1) reported to be an elicitor of plant defenses (Fellbrich et al., 2002). This peptide is required for virulence in barley (Fiegen and Knogge, 2002). We also found a cupin 5 SSP, reportedly involved in oxidative stress reactions (Dunwell, 1998; Cechin et al., 2008). Two upregulated cupredoxins (SSPs, 19,745, and 94,041) could participate in copper stabilization in concert with tyrosinase (574,375 and 308,561) for pigment production and/or with the multi copper-dependent laccase (599,951, CAZyme AA1) for lignin degradation (Benoit et al., 2015). A lactonehydrolase LSP (243,454) was found highly upregulated. Lactonehydrolase was shown to detoxify the zearalenone mycotoxin (Takahashi-Ando et al., 2002). Brown et al (Brown et al., 2012) found two detoxifying lactonehydrolases in *F. graminearum* and hypothesized a self-defense mechanism. Finally, the cysteine-rich SSP cassiicolin, only effector characterized to date in *C. cassiicola* (Déon et al., 2012a,b) was also found among the upregulated SSPs, as expected.

### Other differentially expressed genes

Beside the 92 differentially expressed candidate effectors (Figure 4), 261 other genes were significantly modulated in our RNA-Seq experiment (Table 2, Table S6), evenly distributed between upregulated and downregulated genes (respectively 129 and 132 genes), in contrast with candidate effectors that were mostly upregulated during the interaction with the host.

We carried out GO term enrichment analysis on this set of genes (Table S7). In the downregulated gene cluster, terms associated with transcriptional and translational activities were overrepresented. In the upregulated gene cluster, terms associated with membrane transport (notably of carbohydrates) and oxidoreductase activity were over-represented. We can speculate that the decrease in both transcription and translation of these fungal genes is part of the response to plant defense mechanisms, while the upregulation of transporter activities is in line with increased nutrient acquisition by the pathogen.

Among the genes identified as singletons according to our interspecific protein clustering (Figure 2, Table S2) eleven were differentially expressed (i.e., 93,300, 567,860, 646,750, 119,471, 579,535, 594,582, 641,745, 673,479, 372,273, 349,923, and the cassiicolin gene *Cas1*). However, no annotation was currently available for these, besides cassiicolin and two other genes annotated as encoding secreted proteins (the LSP 673479 and the SSP 646750). Genes of still unknown function, specific to our pathogen and differentially expressed during its compatible interaction with the rubber tree, are clearly of interest and warrant further study.

This transcriptomic analysis has allowed the identification of differentially expressed genes, among which 45 putative secreted candidate effectors may play a key role in this compatible interaction with the rubber tree. This now needs validation by functional analysis.

### PART 3. intraspecific comparative genomics

In the first and second part of this study we identified all putative effectors in the CCP genome and experimentally narrowed the list to those modulated during the compatible interaction with the rubber tree. The third objective of our study was to identify the effectors that could explain specific features in terms of pathogenicity and virulence profiles among the highly diverse *C. cassiicola* isolates. We thus sequenced the genomes of 35 plant-associated *C. cassiicola* isolates (Table 3), using Illumina technology with a theoretical average depth of ~45x, to conduct an intra-species comparison of all putative effectors. The 35 isolates were mostly collected from *Hevea brasiliensis* (74%), but also from other host plants: *Cucumis sativus* (ATI11, CBS129.5, EDIG, IA, JQ, PB), *Glycine max* (777AA, RUD), *Piper hispidinervum* (LP07) and *Vernonia cinerea* (GSO2). All were collected from lesioned leaves, except for three (E55, E79, and E139) which originate from asymptomatic rubber tree leaves from Brazil, where CLF is absent or very limited. We selected members belonging to various phylogenetic clades according to a prior phylogeny based on four concatenated *loci* sequences (Déon et al., 2014). Among each clade, whenever available, we selected isolates with and without cassiicolin genes, with diversified geographical origins. Isolate CCP was used as the reference.

**Table 3:** Resequenced corynespora cassiicola isolates and Whole Genome Shotgun (WGS) sequencing metrics.

**Isolate name**	**Geographical origin**	**Host plant**	**Sanitary status of host tissue**	**^a^Toxin class**	**WGS mapping on CCP**	**WGS** ***de novo*** **assembly**						
					**Properly mapped reads (%)**	**Assembled genome size**	**^b^Contigs**	**^c^N50**	**Used reads**	**Used reads (%)**	**Coverage depth**	**Number of predicted genes**
777AA	Brazil (BR)	*Glycine max* (Gm)	Lesioned	Cas2	69,6	43,337,818	25,946	59,381	20,252,558	95,7	36	16,745
ATI11	Brazil (BR)	*Cucumis sativus* (Cs)	Lesioned	Cas2	69,7	42,232,695	18,580	55,794	18,402,338	96,0	34	17,533
CBS129.25	Brazil (BR)	*Cucumis sativus* (Cs)	Lesioned	Cas2	73,4	41,411,750	11,707	65,420	21,960,627	96,0	43	16,848
CCAM1	Cameroon (CA)	*Hevea brasiliensis* (Hb)	Lesioned	Cas0	81,4	43,770,965	25,726	44,219	15,395,570	96,2	28	20,398
CCAM2	Cameroon (CA)	*Hevea brasiliensis* (Hb)	Lesioned	Cas1	93,7	43,143,070	21,575	45,751	19,761,908	95,3	36	20,926
CCAM3	Cameroon (CA)	*Hevea brasiliensis* (Hb)	Lesioned	Cas1	91,5	44,706,950	34,046	44,020	25,509,053	95,2	43	23,231
CCAM4	Cameroon (CA)	*Hevea brasiliensis* (Hb)	Lesioned	Cas0	82,1	43,735,702	24,864	50,281	23,499,794	96,0	43	20,437
CCI13	Côte d'Ivoire (CI)	*Hevea brasiliensis* (Hb)	Lesioned	Cas0	68	43,462,677	26,299	57,107	20,289,471	95,8	35	17,139
CCI6	Côte d'Ivoire (CI)	*Hevea brasiliensis* (Hb)	Lesioned	Cas0	68,8	43,521,463	25,732	58,564	21,920,208	95,6	38	17,106
**CCP**	**Philippines (PH)**	*Hevea brasiliensis (Hb)*	**Lesioned**	**Cas 1**		**44,850,000**	**244**					**17,167**
CGAB1	Gabon (GA)	*Hevea brasiliensis* (Hb)	Lesioned	Cas0	70,9	42,622,471	23,001	54,421	15,652,205	96,2	29	17,143
CGAB2	Gabon (GA)	*Hevea brasiliensis* (Hb)	Lesioned	Cas0	70	42,753,115	25,233	56,175	15,803,351	96,1	29	17,320
CIND3	India (IN)	*Hevea brasiliensis* (Hb)	Lesioned	Cas0	68,4	43,142,275	25,446	55,647	18,552,642	95,9	33	17,065
CLN16	Malaysia (MA)	*Hevea brasiliensis* (Hb)	Lesioned	Cas0	69,6	43,236,492	26,480	49,745	14,478,070	95,8	26	17,203
CSB16	Malaysia (MA)	*Hevea brasiliensis* (Hb)	Lesioned	Cas5	66,9	41,090,012	8,172	58,209	17,911,142	95,2	34	15,160
CSRI1	Sri Lanka (SL)	*Hevea brasiliensis* (Hb)	Lesioned	Cas0	66,2	41,512,866	16,169	57,706	20,366,586	95,8	39	16,143
CSRI2	Sri Lanka (SL)	*Hevea brasiliensis* (Hb)	Lesioned	Cas5	65,4	42,036 764	22,858	57,382	19,938,115	95,7	37	16,247
CSRI5	Sri Lanka (SL)	*Hevea brasiliensis* (Hb)	Lesioned	Cas0	69	43,093,641	24,753	56,383	19,074,836	95,5	34	17,128
CTHA1	Thaïland (TH)	*Hevea brasiliensis* (Hb)	Lesioned	Cas0	84,4	42,179,887	16,615	44 014	19,483,933	95,7	38	19,069
CTHA2	Thaïland (TH)	*Hevea brasiliensis* (Hb)	Lesioned	Cas0	67	41,852,029	18,777	54 006	19,105,681	96,0	37	16,189
CTHA3	Thaïland (TH)	*Hevea brasiliensis* (Hb)	Lesioned	Cas0	84,2	40,558,447	6,035	33 197	12,008,130	95,5	24	15,705
CTHA4	Thaïland (TH)	*Hevea brasiliensis* (Hb)	Lesioned	Cas0	86,3	40,998,259	6,794	43 846	25,526,040	95,2	51	16,564
CTHA5	Thaïland (TH)	*Hevea brasiliensis* (Hb)	Lesioned	Cas0	71,8	41,979,272	19,267	65 277	15,226,899	95,7	29	17,056
CTHA6	Thaïland (TH)	*Hevea brasiliensis* (Hb)	Lesioned	Cas0	70,6	42,283,119	24,161	72 894	14,104,323	94,8	27	17,145
E139	Brazil (BR)	*Hevea brasiliensis* (Hb)	Healthy	Cas4	80,2	44,431,849	27,829	45,812	23,838,352	96,2	43	21,058
E55	Brazil (BR)	*Hevea brasiliensis* (Hb)	Healthy	Cas0	74,3	41,269,705	16,066	49,888	25,968,811	94,2	48	15,772
E79	Brazil (BR)	*Hevea brasiliensis* (Hb)	Healthy	Cas4	81,2	44,028,261	27,903	47,985	22,067,764	96,2	40	21,866
EDIG	Brazil (BR)	*Cucumis sativus* (Cs)	Lesioned	Cas2	70,5	42,249,625	17,402	37,754	12,130,622	96,2	22	17,357
GSO2	Brazil (BR)	*Vernonia cinerea* (Vc)	Lesioned	Cas2	67,4	43,198,292	22,836	30,770	12,431,866	95,7	22	17,060
IA	Brazil (BR)	*Cucumis sativus (Cs)*	Lesioned	Cas2 + 7	68,7	42,992,747	23,053	58,869	29,543,154	96,0	53	17,059
JQ	Brazil (BR)	*Cucumis sativus* (Cs)	Lesioned	Cas2 + 7	70,5	42,862,184	20,611	55,346	17,233,530	96,1	32	17,001
LPO7	Brazil (BR)	*Piper hispidinervum* (Ph)	Lesioned	Cas0	71,8	43,904,214	30,661	49,813	25,807,090	95,3	47	19,014
PB	Brazil (BR)	*Cucumis sativus* (Cs)	Lesioned	Cas2 + 7	69,4	42,924,591	21,830	55,818	22,793,115	96,1	41	16,977
RUD	Brazil (BR)	*Glycine max* (Gm)	Lesioned	Cas2 + 6	70,8	42,698,174	18,250	49,215	15 754,738	95,8	29	16,905
SS1	Malaysia (MA)	*Hevea brasiliensis* (Hb)	Lesioned	Cas5	66,9	41,929,664	21,219	52,434	17,449,048	95,7	34	16,152
TSB1	Malaysia (MA)	*Hevea brasiliensis* (Hb)	Lesioned	Cas5	66,3	42,091,408	21,882	55,474	20,226,399	95,7	38	16,238

*In bold characters: reference CCP isolate*.

a *Toxin class according to this analysis*.

b *Number of contigs in each genome (scaffolds in the case of CCP)*.

c *Contig length at which 50% of total bases in the assembly are in contigs of that length or greater*.

Our initial strategy was to map the 767 million WGS short reads from the 35 isolates onto the CCP genome assembly. This mapping strategy can reveal structural variations of candidate effectors, such as sequence polymorphism, presence/absence variants (PAVs) and copy number variants (CNVs). However, the mapping rates were found to be too low for most isolates (Table 3), ranging from 65.4 to 93.7% of the reads (73.3% on average). In addition, this strategy failed to reveal the cassiicolin locus in 15 isolates of our selection from which this gene was previously amplified by PCR (Déon et al., 2014). We thus moved on to *de novo* assembly. The proportion of sequence reads used in the assemblies of the 35 genomes (min 94.2%, max 96.2%, average 95.7%) was significantly higher than the proportion of reads mapped onto CCP. The assembled genome sizes are very similar to CCP, ranging from 40 to 44.7 Mb. No correlation was observed between assembled genome size and depth, and so we considered that the sequencing effort was sufficient. We then searched for orthologs of all predicted CCP genes in the different assemblies. We found values ranging from 15,160 to 23,231 genes where CCP had 17,167 genes. No clear relation could be found between genome size or gene number and features such as host or geographical origin.

### Intraspecific genome-wide phylogeny

**Figure 5:**
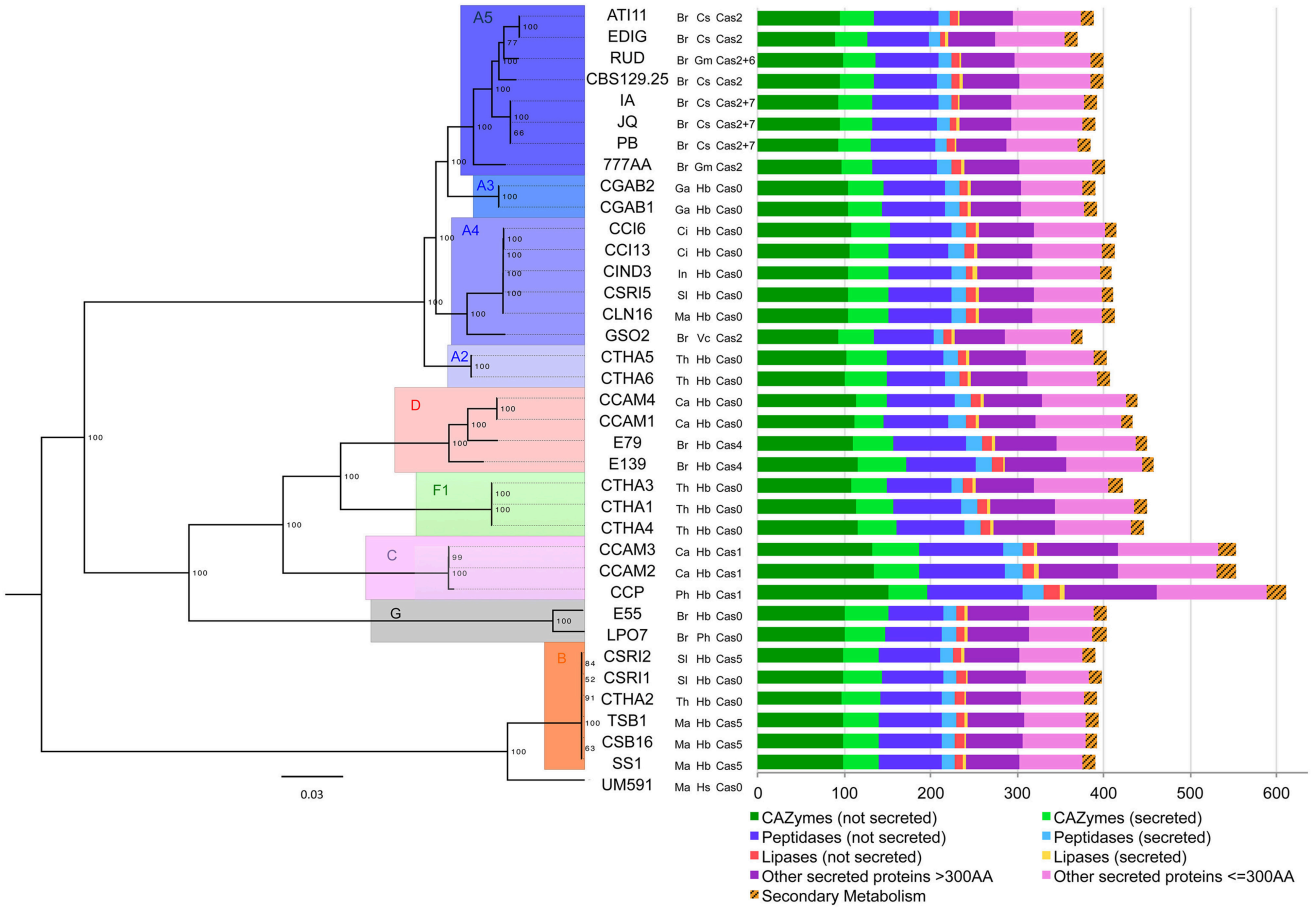
Intraspecific phylogenomic tree and putative accessory effectors of *C. cassiicola* isolates. The maximum likelihood phylogenomic tree was based on 12,420 conserved protein sequences. Branch lengths are indicated by the bar (substitutions/site); 1,000 bootstrap values are shown as percentages. The isolates names are followed by their country and host codes, and by the toxin class, as indicated in Table 1. The diagram on the right represents the composition in putative accessory effectors (i.e. either absent or varying in copy number in at least one isolate).

We built an instraspecific genome-wide phylogeny of 37 *C. cassiicola* isolates (i.e. CCP, the 35 resequenced plant-associated isolates and the human-hosted isolate UM591). The 651-sequence core set previously used for the intraspecific phylogeny was found insufficient to discriminate closely related *C. cassiicola* isolates. We thus used a new core set of 12,112 protein sequences (70% of CCP predicted proteome) found in exactly one copy in each isolate. Overall, this genome-wide intraspecific phylogeny (Figure 5) was in close agreement with the one previously built using four *loci* only (Déon et al., 2014). All previously identified clades and sub-clades were confirmed. Moreover, eight isolates from Clade A (ATI11, EDIG, RUD, CBS129.5, IA, JQ, PB, and 777AA) now formed a new highly supported subclade that was named A5. These were Brazilian isolates from cucumber and soybean, while all other isolates in Clade A originated from the rubber tree (except for GSO2, isolated from *Vernonia*). Several previously unclassified isolates could be positioned: CCAM1 and CCAM4 were placed in Clade D with E139 and E79; LP07 joined E55 in Clade G and GSO2, while slightly apart, was included in Subclade A4. This robust genome-wide phylogeny shows that an early differentiation event occurred between Clade B and the other clades.

UM591was found to be distantly related to plant isolates of Clade B4. Whether UM591 belongs to a distinct Subclade in B or a new clade needs further investigation. Although it shares a common ancestor with plant isolates, there is no current evidence that UM591 is a plant pathogen itself, or conversely that plant isolates could also infect humans.

### *C. cassiicola* core and accessory effectors

We searched for homologs of the 2,870 CCP putative effectors in the 35 re-sequenced genomes of the plant-associated *C. cassiicola* isolates. Among them, 2,258 genes (78.6%) were found in exactly one copy in each isolate and were considered as putative “core effectors” for this set of isolates. The remaining 612, either absent or present in multiple copies in at least one isolate (e.g. *Cas*), were considered as putative “accessory effectors” (Table 4, Table S8). Accessory effectors were found in all functional categories, in variable ratios: 24 were associated with the secondary metabolism (38.1% of all putative effectors in this category), 195 were CAZymes (16.9%), 24 lipases (3.9%), 135 peptidases (22.1%) and 234 “other secreted proteins” divided into 108 LSPs (26.3%) and 126 SSPs (24.7%). The distribution was evenly balanced between secreted (307) and non-secreted (305) accessory effectors. The distribution of the putative accessory effectors in the different isolates is depicted in Figure 5 as the sum of orthologs found in each functional category. CCP isolate, being the reference, has all 612 putative accessory effectors. Of these, only 20 were specific to CCP (1 secondary metabolism gene, 6 CAZymes, 4 peptidases, 1 lipase, 4SSPs, 3 LSPs, Table S8). Twenty-two others, shared by CCP, CCAM2 and CCAM3, were specific to Clade C isolates (2 secondary metabolism genes, 8 CAZymes, 7 peptidases, 1 lipase, 4 LSPs) (Table S8). However, no effector specific to an isolate other than CCP (i.e., not sharing significant homology with a CCP protein) could be identified, since CCP was used as the reference.

**Table 4:** Putative core and accessory effectors identified among 36 *C. cassiicola* isolates.

**Effector categories**	**CCP total effectors**			**Accessory effectors**		
	**Total**	**Secreted**	**Not secreted**	**Total**	**Secreted**	**Not secreted**
				**Nbr (% of CCP)**	**Nbr (% of CCP)**	**Nbr (% of CCP)**
Secondary metabolism	63		63	24 (38.1)		24 (38.1)
Cazymes	1,152	326	826	195 (16.9)	45 (13.8)	150 (18.2)
Lipases	104	40	64	24 (3.9)	5 (12.5)	19 (29.7)
Peptidases	630	124	506	135 (22.1)	23 (18.5)	112 (22.1)
Other LSPs	411	411		108 (26.3)	108 (26.3)	
Other SSPs	510	510		126 (24.7)	126 (24.7)	
Total	2,870	1,411	1,459	612 (21.3)	307 (21.8)	305 (20.9)

*Accessory effectors are effectors either absent or varying in copy number in at least one isolate, as opposed to the core effectors, found in exactly one copy in each of the 36 isolates. Only conventional ER/Golgi-dependent secretion was considered; LSP (large secreted proteins, >300 amino acids), SSP (small secreted proteins, <300 amino acids)*.

**Figure 6:**
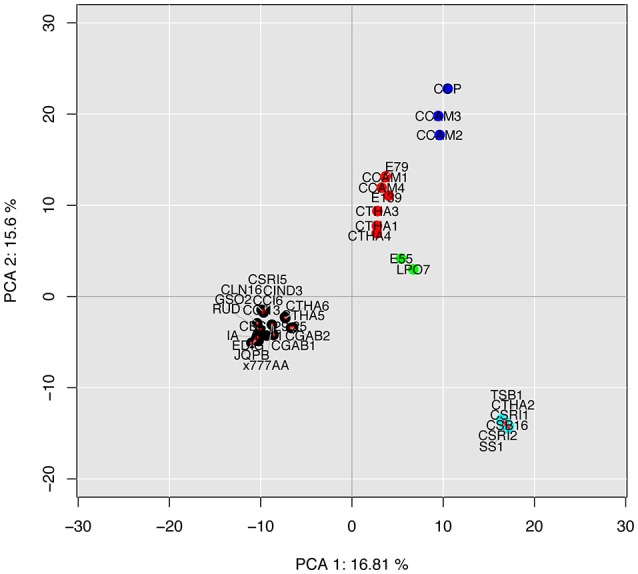
Intraspecific principal component analysis (PCA) of 36 plant-associated *C. cassiicola* isolates based on their composition in putative accessory effectors (i.e. either absent or varying in copy number in at least one isolate). PCA was computed from the counts in each effector subcategory (611 dimensions). Different colors represent different clusters.

We carried out an intraspecific PCA based on the composition in accessory effectors found among the 36 *C. cassiicola* isolates (Figure 6). Despite the high number of dimensions, the first two explained >32% of the total variance. The classification obtained was fully consistent with the genome-wide phylogeny depicted in Figure 5. We note that the phylogram and the accessory effector PCA cladogram were obtained by totally independent datasets: in the first case, the ~12,000 core genes (found in all isolates in one copy), and in the second case, the accessory effector gene subset. This result suggests that new accessory effectors have been acquired mostly through evolution. However, isolated acquisitions by horizontal gene transfer cannot be excluded, though not detected here. Whatever the classification approach, no clear structure by geographic origin or host plant could be established.

Further work could seek to relate specific sets of predicted effectors with biological features such as pathogenicity specificities on various hosts, or virulence profiles on different rubber clones. However, such biological information is still scarce. CCP and its closest relatives, CCAM2 and CCAM3 (Clade C), show the highest effectors diversity. They were previously found to be more aggressive than isolates of other types when spore-inoculated on the susceptible rubber clone PB217 (Déon et al., 2014). Their filtrates were also found to be highly toxic when tested on detached leaves over a range of rubber clones, using a method based on electrolyte-leakage measurements (Tran et al., 2016). We advance the hypothesis that the effector expansion in Clade C isolates may favor higher virulence and/or a broader host range. Some isolates in Clade A4 also displayed high filtrate toxicity on various clones in Africa (Tran et al., 2016). In our analysis, four putative accessory effectors (626988, 580294, 580296 and 580661) were found in all A4 isolates, but not in other types besides two Clade C and two Clade A5 isolates. None was differentially expressed in CCP, but their expression in A4 isolates remains to be investigated. Isolates in Clade B are also frequently associated with CLF in Asia (Shuib et al., 2015). In our study, nine candidate effectors, all potentially secreted, were shared by Clade B and C isolates only, among which one LSP (577878) differentially expressed in CCP. Finally, three isolates (E55, E79 and E139) categorized as rubber tree endophytes (Déon et al., 2012b) had effector repertoires equivalent to or even larger than that of pathogenic isolates of the same clades. These endophytic isolates may be adapted to a broader host range thanks to their large effector repertoires. Whether they may be necrotrophs on some hosts and which effector(s) might be involved is unknown.

Within the 353 differentially transcribed CCP genes revealed by the RNA-Seq analysis (Table S6), 19 were categorized as potential accessory effectors (Table S8). Fourteen were upregulated and five downregulated. Nine are CAZymes (597,988; 577,841; 574,440; 577,285; 568,400; 569,191; 577,402; 618,747; 600,578), four are peptidases (149614; 495460; 366051; 514786), three are “other LSPs” (513,290; 673,479; 577,878) and three are up-regulated SSPs, among which cassiicolin, shared by 18 isolates, and two uncharacterized SSPs (24,345 and 646,750), specific to CCP.

The interspecific clustering revealed 4,047 singletons in the CCP genome (i.e. without homology in the other species) (Table S2), of which 158 were putative effectors. Our intraspecific analysis revealed that 29 of them were accessory, including two associated with the secondary metabolism, five CAZymes, five lipases, one peptidase, nine SSPs and seven LSPs (Table S8). Interestingly, the SSP 646750 discussed above is the only putative accessory effector differentially expressed (upregulated) in the compatible interaction that has no ortholog at either the interspecific or intraspecific scale. However, its specificity to the CCP isolate could be artefactual, due to the lack of assembly of the genome region carrying this gene.

### PART 4: the case of cassiicolin

The most emblematic and best characterized *C. cassiicola* effector to date is the cassiicolin toxin. The *Cas1* gene (GenBank EF667973) was not predicted by the JGI annotation pipeline despite high RNA-Seq reading coverage (>3,900), possibly due to its short size and its intron-exon organization. We could nevertheless locate it on scaffold 130:2337-2848, minus strand. According to the JGI VISTA plot of scaffold 130 (4,131 bp), compared with the other genomes of our interspecific study, no nucleotide sequence homology could be found by blast against the entire NCBI nr and MycoCosm databases. This suggests that the genomic region bearing *Cas1* was acquired recently, either through evolution or horizontal gene transfer from still unknown organisms. Interestingly, a fragment homologous to a MOLLY transposon from *S. nodurum* (GeneBank AJ488502) was located at the end of scaffold 130, only ~2 kb downstream from *Ca1*. Another gene (580614) encoding a predicted protein with a SAM-methyltransferase conserved domain was identified on the same scaffold, between *Cas1* and the MOLLY transposon sequence. Proximity with the transposon suggests that *Cas1* and neighboring genes may have been multiplied through transposition events, although draft genome assembly alone could not support this hypothesis. The very high RNA-Seq reads coverage of *Cas1* (>3,900) supports this hypothesis. The neighboring SAM-methyltransferase gene may be involved in the regulation of cassiicolin activity through methylation: the mature cassiicolin was shown to carry a methylated mannose on the second residue (Barthe et al., 2007), but whether this post-transcriptional modification is required for virulence is unknown.

In this study, we could confirm that the cassiicolin gene is transcriptionally up-regulated during the early phase of the compatible interaction with the rubber tree (Figure 4). PCR screening had previously evidenced that it was an accessory effector, since it was detected in only 47% of the tested *C. cassiicola* isolates, with various isoforms (Cas1 to Cas6) used to delineate toxin classes (Déon et al., 2014). Our intraspecific comparative genomic analysis confirmed the previously defined toxin classes for most isolates except IA, JQ and PB, three phylogenetically close relatives isolated from cucumber (Figure 5). A *Cas2* gene was detected in isolate JQ, previously categorized as Cas0. Additionally, we were able to identify a new cassiicolin isoform, shared by IA, JQ and PB, that was named Cas7. To rule out any artifact due to the assembly process, we confirmed the sequence by PCR amplification and Sanger sequencing of that region. The *Cas7* sequence was registered in GenBank (BankIt2035475 Seq1 MF564202). It is quite divergent from the previously identified cassicolin genes, with only 72.3–76.7% nucleotide sequence identity and 71.9–77.8% deduced amino acid sequence identity. However, we were not able to amplify *Cas7* transcripts from germinating spores of the Cas2+7 isolates IA, JQ and PB. RT-qPCR analysis of Cas2+7 isolates during interactions with plant hosts may demonstrate the functionality of *Cas7* and confirm its protein sequence.

Cassicolin is the perfect illustration of a species-specific effector responsible for intraspecific diversity due to sequence polymorphism, presence/absence and copy number variations. However, the phenotypic traits associated with these variations are still unclear. Our transcriptomic analysis revealed the existence of other candidate effectors with similar features, *i.e*. secreted proteins differentially expressed during the compatible interaction between *C. cassicola* isolate CCP and the rubber tree, with similar or higher orders of fold change magnitude. Further experimentation will thus be needed to clarify the role of each candidate in CLF.

## General conclusion

This study provides the genomic description of a plant pathogenic *C. cassiicola* isolate and the first transcriptomic analysis for this species. By combining *in silico* mining of all putative effectors and experimental identification of the fungal genes differentially expressed during the compatible interaction with the rubber tree, we were able to identify pertinent candidate effectors potentially involved in CLF disease, in addition to the already characterized effector cassiicolin. Resequencing and *de novo* assembly of a large set of *C. cassiicola* isolates allowed very accurate refining of the intraspecific phylogeny and the genome-wide description of their respective effector repertoires. In addition to core effectors shared by all isolates, accessory effector repertoires were generally consistent with the phylogeny, besides a few isolate-specific variations. At this stage, it is difficult to offer a cogent interpretation of such specificities owing to the lack of phenotypic information in terms of virulence profiles or host ranges. Further transcriptomic, proteomic or metabolomic analyses may help identify the most pertinent effectors. These could then be used to develop phenotyping tests or genotyping tools, after identification of their plant molecular targets, for the selection of tolerant cultivars.

## Author contributions

VP-R conceived and managed the research, with scientific support from FM. DL performed data mining of the reference genome, assemblies of the newly sequenced genomes, inter- and intraspecific comparative genome analyses, and RNAseq analyses. SR managed the biological material and performed the plant-pathogen interaction experiment. DL and SR performed the nucleic acid extractions. FM and AK mediated the collaboration with DOE-JGI and provided expertise for the interspecific analysis. VP-R and RdO provided isolates from rubber tree and other hosts respectively. PL built and managed the RNAseq pipeline used in Clermont-Ferrand. BF provided expertise in phylogeny. J-SV provided expertise for the manual tuning of clustering parameters. KuL, AL, KaL, DB, RO, and KB, performed sequencing, assembly and annotation of the reference genome at DOE-JGI. JS and IG managed the 1,000 Fungal Genomes Project. DL and VP-R wrote the draft article. All authors read, corrected and approved the article.

### Conflict of interest statement

The authors declare that the research was conducted in the absence of any commercial or financial relationships that could be construed as a potential conflict of interest.
