# Supplementary figures and images for: Genome-Wide Analysis of Corynespora cassiicola Leaf Fall Disease Putative Effectors
Source: Front Microbiol. 2018 Mar 2;9:276. doi: 10.3389/fmicb.2018.00276 (PMC5840194; doi:10.3389/fmicb.2018.00276)

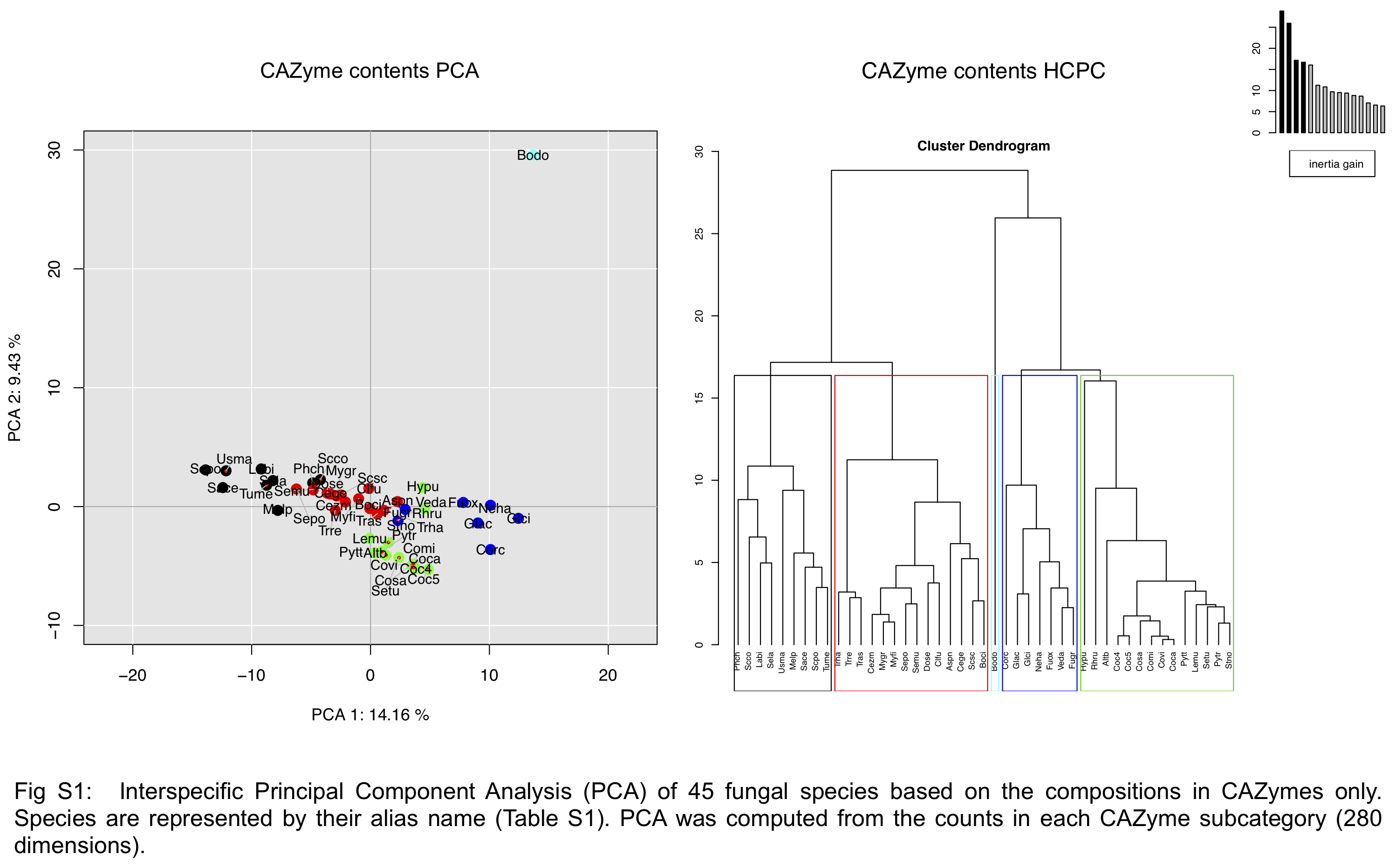

Supplement: Supplementary file 9 [file Image1.TIF]

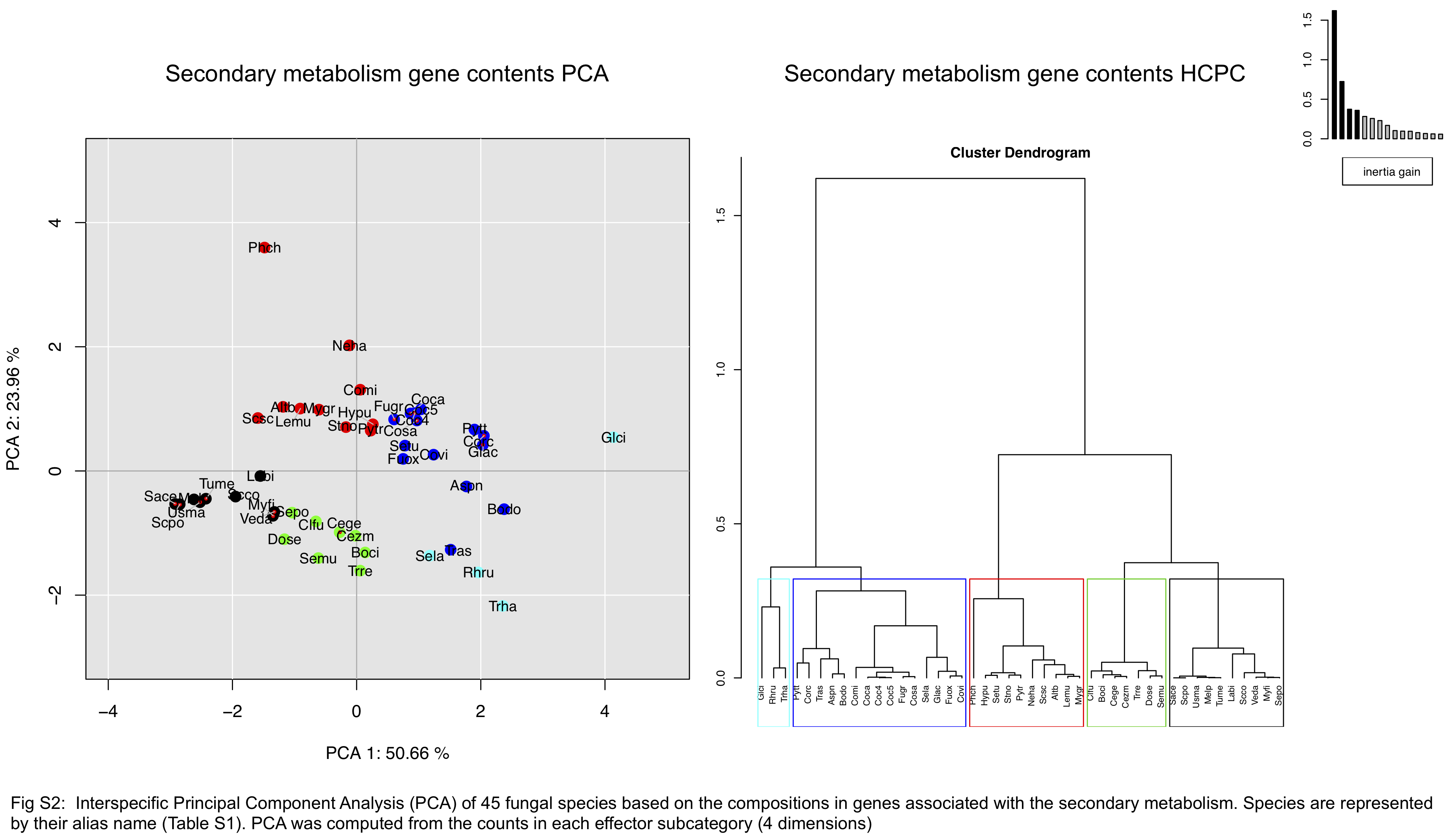

Supplement: Supplementary file 10 [file Image2.TIF]
